# Dispensing Processes Impact Apparent Biological Activity as Determined by Computational and Statistical Analyses

**DOI:** 10.1371/journal.pone.0062325

**Published:** 2013-05-01

**Authors:** Sean Ekins, Joe Olechno, Antony J. Williams

**Affiliations:** 1 Collaborations in Chemistry, Fuquay-Varina, North Carolina, United States of America; 2 Labcyte Inc., Sunnyvale, California, United States of America; 3 Royal Society of Chemistry, Wake Forest, North Carolina, United States of America; UMR-S665, INSERM, Université Paris Diderot, INTS, France

## Abstract

Dispensing and dilution processes may profoundly influence estimates of biological activity of compounds. Published data show Ephrin type-B receptor 4 IC_50_ values obtained via tip-based serial dilution and dispensing versus acoustic dispensing with direct dilution differ by orders of magnitude with no correlation or ranking of datasets. We generated computational 3D pharmacophores based on data derived by both acoustic and tip-based transfer. The computed pharmacophores differ significantly depending upon dispensing and dilution methods. The acoustic dispensing-derived pharmacophore correctly identified active compounds in a subsequent test set where the tip-based method failed. Data from acoustic dispensing generates a pharmacophore containing two hydrophobic features, one hydrogen bond donor and one hydrogen bond acceptor. This is consistent with X-ray crystallography studies of ligand-protein interactions and automatically generated pharmacophores derived from this structural data. In contrast, the tip-based data suggest a pharmacophore with two hydrogen bond acceptors, one hydrogen bond donor and no hydrophobic features. This pharmacophore is inconsistent with the X-ray crystallographic studies and automatically generated pharmacophores. In short, traditional dispensing processes are another important source of error in high-throughput screening that impacts computational and statistical analyses. These findings have far-reaching implications in biological research.

## Introduction

There have been many studies which have evaluated aspects of biological assays and the tools involved which could result in errors and erroneous data. Processes like tip-based and acoustic dispensing have a profound influence on estimates of compound activity. Several independent studies of high-throughput screening (HTS) show that the two techniques generate conflicting results [1], [2], [3], [4], [5]. The difference in results may mean missing important lead compounds, following dead-ends and developing inappropriate compounds for activity optimization.

Previous research has impugned tip-based techniques because they can generate errors due to leachates from the plastic that may profoundly affect biological assays [6], [7], [8], [9], [10], [11]. Broadly speaking, the IC_50_ values derived using tip-based serial dilution and dispensing tend to be greater (i.e., show lower potency) than IC_50_ values derived using acoustic dispensing. Some compounds appeared hundreds of times more active with the acoustic process [1], [2], [3], [4]. We now address how these errors may affect computational models and propagate poor data in both proprietary and public databases, the result of which is likely to misdirect drug design.

While we are limited by the number of compounds available with data in tip-based and acoustic dispensing, this study suggests a significant impact on drug design, especially when coupled with other reports of poorly correlating IC_50_ results in which larger number of molecules are used but the molecular structures are not provided for computational analysis [1], [2], [12]. We now show how dispensing processes impact computational and statistical results.

## Materials and Methods

### Dataset

**Figure 1:**
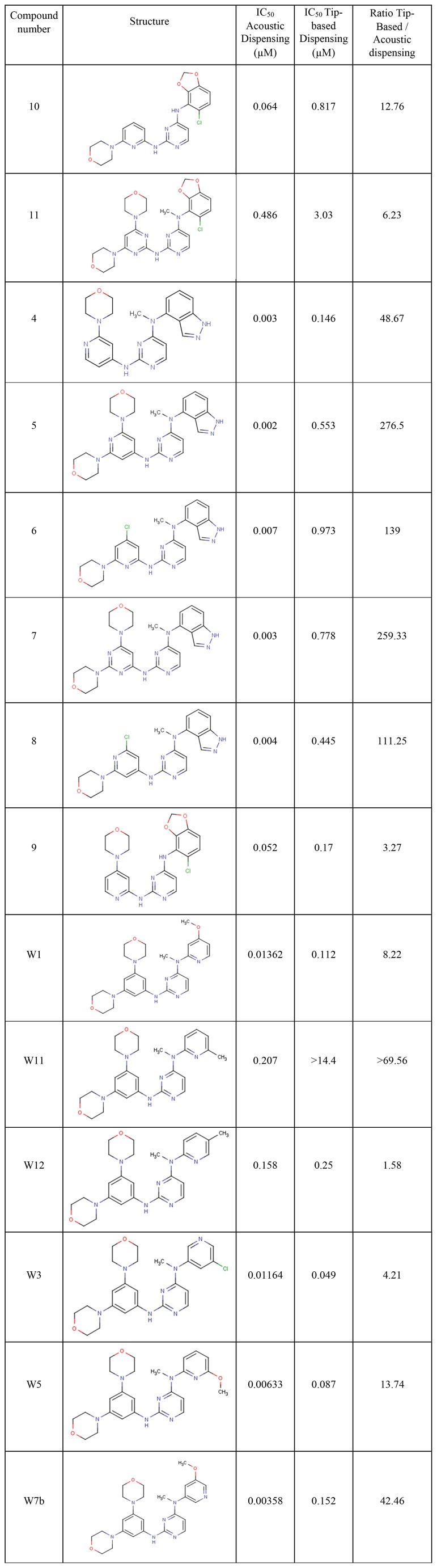
The EphB4 IC_50_ values produced via acoustic transfer with direct dilution are significantly lower (more biologically active) than when generated with tip-based transfer and serial dilutions. The ratio of the EphB4 IC_50_ values varies widely and correlates poorly with the calculated logP of the compounds (see also Table S1). The compounds and data were published in patents by AstraZeneca [19], [20].

This paper is based on the published comparisons of IC_50_ values determined by AstraZeneca scientists [19], [20] (Fig 1) for inhibition against the Ephrin type-B receptor 4 (EphB4), a membrane-bound receptor tyrosine kinase that binds to ephrin-B2 ligands bound to the surfaces of other cells to induce angiogenic events. Unique to these publications, the researchers provided structures of the inhibitors as well as IC_50_ values using both serial dilution facilitated by tip-based dispensing (Genesis, Tecan Ltd, Weymouth, United Kingdom) and direct dilution [26], [27] with an acoustic dispensing system (Echo550, Labcyte Inc., Sunnyvale, CA). They found that the IC_50_ values obtained with acoustic transfers suggested that the compounds were 1.5 to 276.5 times more active than when tip-based techniques were used [19], [20].

### Statistical and Computational Modeling

We used these published data [19], [20] to develop computational pharmacophores and to address correlations of activity with physical properties with commercially available tools.

### Statistical analyses

IC_50_ values (Fig 1, Table S1) derived by each method were initially used to correlate 9 molecular descriptors (molecular weight (MW), calculated logP (LogP), number of hydrogen bond donors (HBD), number of hydrogen bond acceptors (HBA), molar refractivity (MR), polar surface area (PSA), LogD, pH 7, charge at pH 7 and isoelectric point (pI, Table S1 and Table 1), all calculated with MarvinSketch 5.9.3, (ChemAxon, Budapest, Hungary) [28] using SAS JMP (v8.0.1, SAS, Cary, NC). Statistical significance was determined by ANOVA.

**Table 1:** Statistical analysis results for correlations with IC_50_.

Descriptor	Acoustic N = 14	Acoustic N = 16	Tip-based N = 14	Tip-based N = 24
MW	0.03	0.00	0.02	0.06
LogP	0.34*	0.11	0.16	0.39*
HBD	0.18	0.13	0.00	0.07
HBA	0.00	0.01	0.05	0.03
MR	0.00	0.04	0.01	0.01
PSA	0.07	0.04	0.06	0.04
logD pH 7	0.23	0.31*	0.07	0.24*
Charge pH 7	0.04	0.13	0.00	0.00
PI	0.14	0.22	0.07	0.04

Note the correlation of LogP with 14 molecules using acoustic dispensing and how addition of more compounds results in correlations of LogP and LogD with tip-based dispensing. No correlations were observed for EphB4 IC_50_ value against molecular weight, hydrogen bond donors, hydrogen bond acceptors, isoelectric point, polar surface area, molar refractivity or analyte charge. * p<0.05 using ANOVA.

### Pharmacophore models

A 3D pharmacophore was developed with IC_50_ values as the indicator of biological activity. In the 3D pharmacophore modeling approach using Discovery Studio (Accelrys version 2.5.5. San Diego, CA, described previously [29]), ten hypotheses were generated using hydrophobic, HBA, HBD, and the positive and negative ionizable features, and the CAESAR algorithm [30] was applied to the molecular data set (maximum of 255 conformations per molecule and maximum energy of 20 kcal/mol) to generate conformers. The pharmcophore hypothesis with the lowest energy cost was selected for further analysis as this model possessed features representative of all the hypotheses. The quality of the structure-activity correlation between the predicted and observed activity values was estimated using the calculated correlation coefficient (r).

### Pharmacophore testing

After the two different pharmacophores were developed based on the original 14 compounds, we found an additional patent from AstraZeneca that provided the IC_50_ values for an additional 12 compounds. None of these compounds were evaluated using both liquid handling techniques. Ten of the compounds had data based upon tip-based dispensing with serial dilution and two had data based upon acoustic dispensing and direct dilution (Table S1).

### Receptor-Ligand Pharmacophores

Pharmacophores for the tyrosine kinase EphB4 were generated from crystal structures in the protein data bank PDB. Pharmacophores were constructed using the receptor-ligand pharmacophore generation protocol in Discovery Studio version 3.5.5 (Accelrys, San Diego, CA) with minimum features (3) and maximum features (6) as are described elsewhere [18].

## Results

### Statistical analysis

The correlation between the 14 Ephrin type-B receptor 4 log IC_50_ values obtained via tip-based serial dilution and dispensing versus acoustic dispensing with direct dilution is poor (R^2^ = 0.25, Fig S1). The red diagonal line indicates where the values would be if the two methods were equivalent. Note that the IC_50_ values for all 14 compounds were lower (more potent) when acoustic transfer was used. Upon statistical analysis of the 14 IC_50_ values for the two dispensing techniques (Fig 1, Table S1), calculated LogP showed a low but statistically significant correlation with log IC_50_ data for acoustic dispensing (r^2^ = 0.34, p<0.05, N = 14, Table 1). Acoustic dispensing IC_50_ data did not demonstrate a statistically significant ranking of tip-based dispensing data based on Spearman's rho analysis (data not shown). This would suggest that there is no statistically significant correlation or ranking between these two measures. That is, the data generated from the two techniques would lead researchers in completely different directions.

### Computational Pharmacophore Modeling

**Figure 2:**
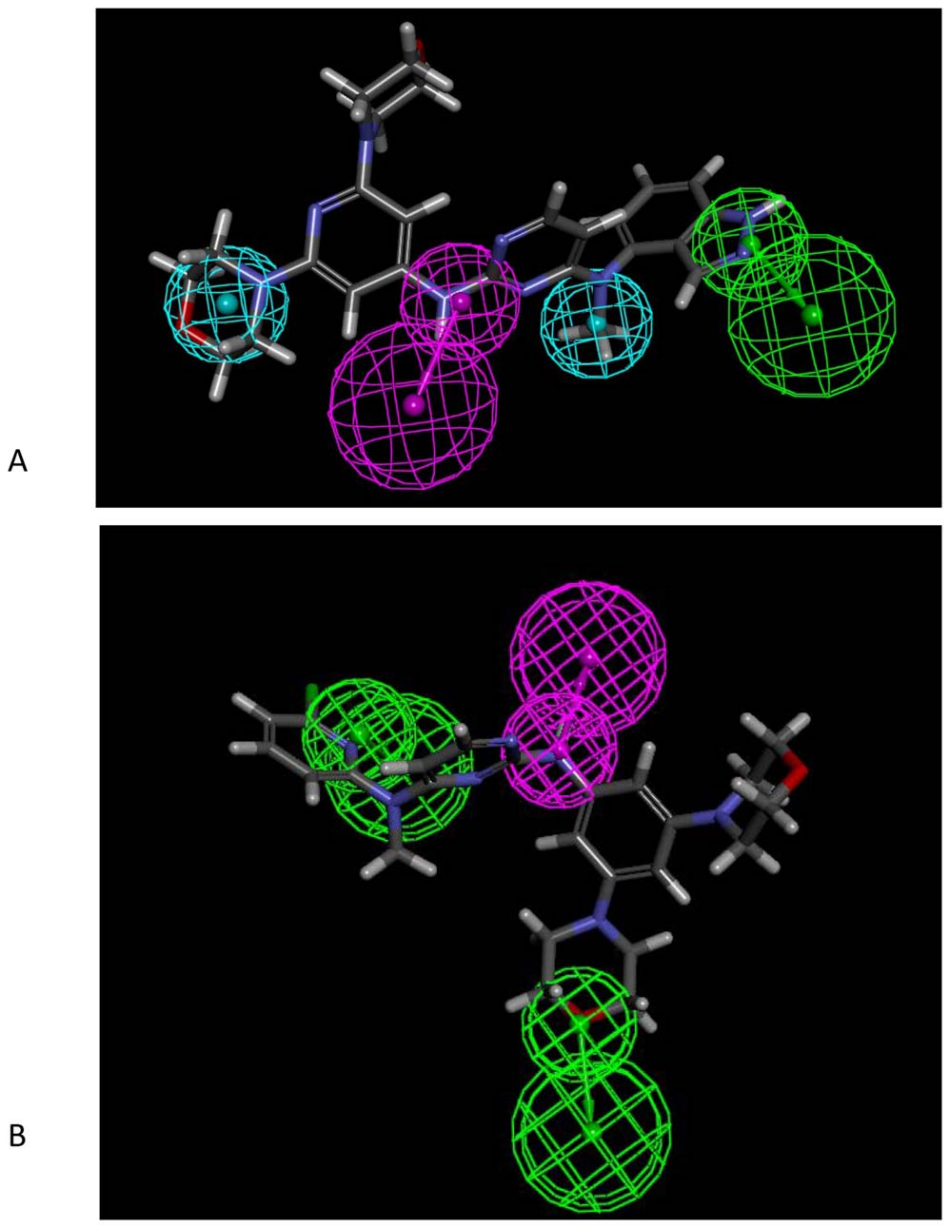
EphB4 ligand-based pharmacophores. (A) EphB4 pharmacophore derived using acoustic dispensing with 14 compounds. The most active compound (5, S1) is shown mapped. (B) EphB4 pharmacophore derived using tip-based dispensing with 14 compounds. The most active compound (w3, S1) is shown mapped. The pharmacophore features are hydrophobic (cyan), hydrogen bond donor (purple) and hydrogen bond acceptor (green).

The pharmacophores (Fig 2A, 2B) derived from data in Table 1 illustrates how the two techniques differ qualitatively. The correlation (r) between predicted and observed IC_50_ values for the pharmacophore derived via acoustic processes was higher than that for the pharmacophore derived from the tip-based processes (Table 2, Results S1). The pharmacophore derived from data generated via the tip-based process also failed, as discussed below, to identify hydrophobic features that were identified in X-ray crystallography 13,14,15,16,17. These hydrophobic features were only evident in the pharmacophore developed through the acoustic transfer process.

**Table 2:** The best EphB4 ligand-based pharmacophore derived from acoustic dispensing data included hydrophobic features not predicted by the tip-based method.

	Hydrophobic features (H)	Hydrogen bond acceptor (HBA)	Hydrogen bond donor (HBD)	Observed vs. predicted IC_50_ r
Acoustic mediated process	2	1	1	0.92
Tip-based mediated process	0	2	1	0.80

The tip-based technique also suggested greater reliance on hydrogen bonding. The ligand-based pharmacophore for the acoustic-based technique showed better correlation than did the pharmacophore developed from tip-based data.

### Pharmacophore testing

The pharmacophores specific to the tip-based and acoustic-based processes were used to predict affinity to EphB4 (as measured by IC_50_ values) for these additional 12 compounds. We used the data to create a 3D multiple conformer database. This database was searched by the two pharmacophores in order to test whether they could discriminate between those with high and low affinity (IC_50_ values). That is, the two pharmacophores developed on the comparative data of 14 compounds were used to test an additional 12 compounds to see whether either of the two pharmacophores developed could predict which compounds in the second set of 12 were most active.

Using the pharmacophore derived via acoustic processes, the two compounds analyzed were predicted to be potent inhibitors. Both of these were compounds that were transferred acoustically. The IC_50_ values actually determined placed these two compounds in the top 3 active compounds (Table S2) and correctly predicted their ranking. The tip-based pharmacophore failed to rank the retrieved compounds correctly (Table S3). This suggests that the pharmacophore developed with tip-based transfers is not useful in predicting the potency of subsequently developed molecules, while the pharmacophore developed with the acoustic procedure is preferred at predicting the activity of new compounds.

When the physical properties of the additional 12 compounds [17] were used in the statistical analysis the calculated LogP and logD showed low but statistically significant correlations with tip-based dispensing (r^2^ = 0.39 p<0.05 and 0.24 p<0.05, respectively, Table 1). This suggests that more data is required in order to observe the importance of hydrophobicity as correlating with tip-based dispensing IC_50_, as previously had been seen with just 14 compounds when using acoustic dispensing but would require significantly more compounds and analyses to be recognized with tip-based dispensing. It is also noted that the hydrophobic features predicted with data generated from acoustic transfers are localized in specific areas and not just a generic increase in hydrophobicity (as measured by LogP, which one might logically expect to lead to greater *non-specific* binding).

### Receptor-Ligand Pharmacophores

**Figure 3:**
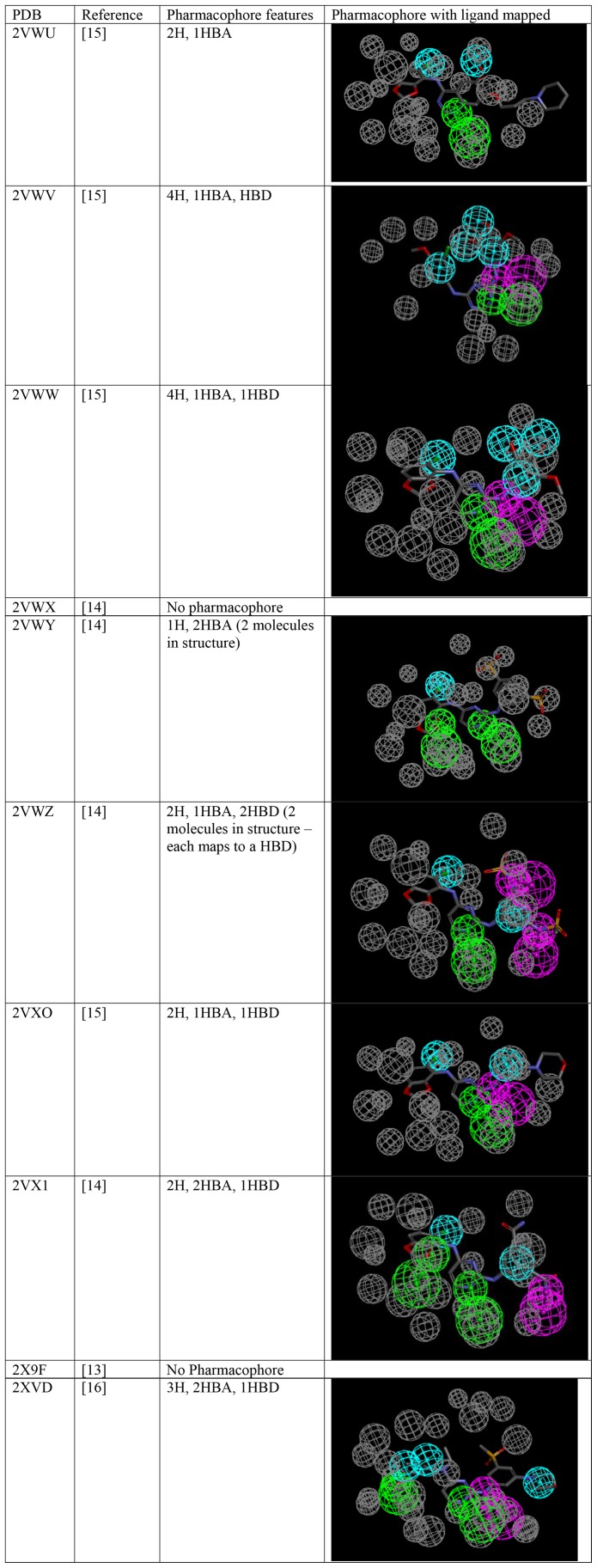
Pharmacophores for the tyrosine kinase EphB4 generated from crystal structures in the protein data bank (PDB). Pharmacophore features are Hydrophobic (H, cyan), Hydrogen bond acceptor (HBA, green) and hydrogen bond donor (HBD, purple). Excluded volumes (grey) were also automatically added.

Receptor-ligand pharmacophores were created in 8 out of 10 cases and all consisted of hydrophobic and hydrogen bonding features (Fig 3).

## Discussion

The pharmacophores derived for the tyrosine kinase EphB4 are dramatically different based upon the process used to set up the dose-response experiments. The pharmacophore derived from the acoustic dispensing data suggests the importance of specific regions of hydrophobicity as well as hydrogen bonding features. The pharmacophore from the tip-derived data suggests only hydrogen bonding features as leading to binding, with no hydrophobic interactions.

In order to further understand the impact of these models, a series of another 4 papers published by AstraZeneca describing structure-based design of tyrosine kinase EphB4 inhibitors were reviewed [13], [14], [15], [16]. These show inhibitors in which part of the molecules are buried in a hydrophobic selectivity pocket beyond Thr693, which appears important for potency. Also indicated were interactions between the inhibitor and Met696 of EphB4 via a hydrogen bond, or acceptor-donor pair. The hydrophobic binding pocket, shown as important for potency by these structure-based studies, was indicated by the acoustic dispensing method in the previous experiments. Interestingly the indazole ring (Fig 2A) has a hydrogen bond acceptor feature which was also noted in the crystal structure of similar compounds [13], suggesting that the acoustic dispensing derived pharmacophore is more representative of the crystal structure data. It should be noted these pharmacophores were derived solely from *in vitro* data of the original articles and not using the crystal structures of the latter work.

We have also used an automated receptor-ligand pharmacophore generation method [18] with the 10 current crystal structures, in order to compare with our initial *in vitro* data ligand pharmacophores further. Receptor-ligand pharmacophores could be created in 8 out of 10 cases and all consisted of hydrophobic and hydrogen bonding features (Fig 3). No pharmacophore was identical to those generated from *in vitro* data alone, however none consisted of solely hydrogen bonding features as in the case of the pharmacophore generated from data using tip-based dispensing (Fig 2B). It is clear that the reported EphB4 kinase inhibitor-crystal structure interactions [13], [14], [15], [16] most closely reflects the pharmacophore derived with the acoustic dispensing data based on independent ligand-dependent pharmacophores, receptor-ligand pharmacophores and statistical approaches taken in this study.

In this study acoustically-derived IC_50_ values were 1.5 to 276.5-fold lower than for tip-based dispensing [19], [20]. Our analyses suggest for the first time that not only are the IC_50_ values unequal but that the data generated by either liquid handling process neither correlates nor, indeed, ranks each other. While the dataset is small it is representative of larger comparisons between dispensing methods that show limited, if any, correlation between IC_50_ results obtained via acoustic transfer and those obtained by tip-based methods [1], [2], [12], [21] (Table 3). No previous publication has analyzed or compared such data (based on tip-based and acoustic dispensing) using computational or statistical approaches. This analysis is only possible in this study because there is data for both dispensing approaches for the compounds in the patents from AstraZeneca that includes molecule structures. We have taken advantage of this small but valuable dataset to perform the analyses described. Unfortunately it is unlikely that a major pharmaceutical company will release 100's or 1000's of compounds with molecule structures and data using different dispensing methods to enable a large scale comparison, simply because it would require exposing confidential structures. To date there are only scatter plots on posters and in papers as we have referenced (Table 3), and critically, none of these groups have reported the effect of molecular properties on these differences between dispensing methods.

**Table 3:** Published comparisons of acoustic and tip-based dispensing.

Dataset/target	Number of molecules	Comments	Reference
12 point IC_50_ values for unspecified dataset	∼40	Compounds more active when using acoustic dispensing. Correlation in data is poor with many compounds showing >10 fold shift in potency depending on dispensing method. No analysis of molecule properties.	[2]
20 point assay IC_50_ values for 5-HT_2b_ agonists	4	3 out of 4 compounds show 5-7 fold increased activity upon acoustic dispensing. No correlation described or analysis of molecule properties.	[21]
20 point assay EC_50_ values for 5-HT_2b_ antagonists	4	3 out of 4 compounds show lower activity upon acoustic dispensing. No correlation described or analysis of molecule properties.	[21]
10 point IC_50_ curves–No target specified	1090	Data appears randomly scattered. 24% had IC_50_ values >3 fold weaker using tip-based dispensing. 8% produced no value using tip-based dispensing. No analysis of molecule properties.	[1]
Inhibition of tyrosine kinases	10,000	Inhibition of reaction was measured at one concentration (10 µM). False positives from acoustic transfer (as measured by subsequent IC_50_ analyses) accounted for 19% of hits. False hits from tip-based transfers accounted for 55% of all hits. 60 more compounds were identified as active with acoustic transfer. No analysis of molecule properties.	[12]

We believe our observations are novel for three reasons. First, no previous publication has shown how data quality can be impacted by dispensing and how this in turn affects computational models and downstream decision making. Second, there has been no comparison of pharmacophores generated from acoustic dispensing and tip-based dispensing. Third, there has been no previous comparison of pharmacophores generated from *in vitro* data with pharmacophores automatically generated from X-ray crystal conformations of inhibitors bound to receptors. We believe our insights to be highly novel and use different technologies to analyze data that cuts across different fields.

## Conclusions

In the absence of structural data, pharmacophores and other computational and statistical models are used to guide medicinal chemistry [22]. Our findings suggest acoustic dispensing methods could improve HTS results and avoid the development of misleading computational models and statistical relationships. While we recently described the errors reported across various internet databases used for biomedical research [23], [24], there has been no analysis of the influence of dispensing processes on such data. It would appear that tip-based dispensing is producing erroneous data based on our and other (Table 3) analyses which we see here reflected in the models and initial lack of correlations with molecular properties. We therefore request that public databases annotate this meta-data alongside biological data points, to create larger datasets for eventually comparing different computational methods in future [25]. This may also assist in the generation of better computational and statistical models from published data. Scientists should be made aware of such dispensing issues and it is therefore important that such evaluations (however limited in molecule numbers) are made accessible for them to decide what technologies to use for dispensing. Such efforts should also encourage pharmaceutical companies to make their data available but we are under no illusions that this will only happen at their convenience e.g. when patents have issued.

## Supporting Information

Figure S1 **A graph of the log IC_50_ values for tip-based serial dilution and dispensing versus acoustic dispensing with direct dilution shows a poor correlation between techniques (R^2^ = 0.246).**
(DOCX)

Results S1 **Showing pharmacophore model information for acoustic-based liquid handling with direct dilution and tip-based liquid handling with serial dilution.**
(DOCX)

Table S1 **Molecule structures, IC_50_ data and descriptors.**
(PDF)

Table S2 **Test set data for searching with ‘acoustic dispensing’ pharmacophore.**
(DOCX)

Table S3 **Test set data for searching with ‘tip-based dispensing’ pharmacophore.**
(DOCX)
