# Supplementary material for: Dispensing Processes Impact Apparent Biological Activity as Determined by Computational and Statistical Analyses
Source: PLoS One. 2013 May 1;8(5):e62325. doi: 10.1371/journal.pone.0062325 (PMC3641061; doi:10.1371/journal.pone.0062325)
Supplement: Figure S1 — A graph of the log IC50 values for tip-based serial dilution and dispensing versus acoustic dispensing with direct dilution shows a poor correlation between techniques (R2 = 0.246). (DOCX) [file pone.0062325.s001.docx]

**Supplemental Data**

**Dispensing Processes Impact Apparent Biological Activity as Determined by Computational and Statistical Analyses**

*Sean Ekins^*1^, Joe Olechno^2^ and Antony J. Williams^3^*

^1^ Collaborations in Chemistry, 5616 Hilltop Needmore Road, Fuquay-Varina, NC 27526, U.S.A.

^2^ Labcyte Inc., 1190 Borregas Avenue, Sunnyvale, CA 94089, U.S.A.

^3^ Royal Society of Chemistry, 904 Tamaras Circle, Wake Forest, NC 27587, U.S.A.

**Figure S1**. A graph of the log IC_50_ (µM) values for tip-based serial dilution and dispensing versus acoustic dispensing with direct dilution shows a poor correlation between techniques (R^2^ = 0.246). The diagonal red line indicates the expected correlation if the two techniques were equivalent. All values are above the diagonal indicating that in this case, the acoustic technique always gave a lower (more potent) IC_50_ value.
