# Supplementary material for: Dispensing Processes Impact Apparent Biological Activity as Determined by Computational and Statistical Analyses
Source: PLoS One. 2013 May 1;8(5):e62325. doi: 10.1371/journal.pone.0062325 (PMC3641061; doi:10.1371/journal.pone.0062325)
Supplement: Results S1 — Showing pharmacophore model information for acoustic-based liquid handling with direct dilution and tip-based liquid handling with serial dilution. (DOCX) [file pone.0062325.s002.docx]

**Supplemental Data**

**Dispensing Processes Impact Apparent Biological Activity as Determined by Computational and Statistical Analyses**

*Sean Ekins^*1^, Joe Olechno^2^ and Antony J. Williams^3^*

^1^ Collaborations in Chemistry, 5616 Hilltop Needmore Road, Fuquay-Varina, NC 27526, U.S.A.

^2^ Labcyte Inc., 1190 Borregas Avenue, Sunnyvale, CA 94089, U.S.A.

^3^ Royal Society of Chemistry, 904 Tamaras Circle, Wake Forest, NC 27587, U.S.A.

**Supplemental Results**

Showing pharmacophore model information for acoustic-based liquid handling with direct dilution and tip-based liquid handling with serial dilution.

Pharmacophore information developed using Accelrys Discovery Studio (v2.5.5. San Diego, CA) for each dataset.

Acoustic-based liquid handling with direct dilution


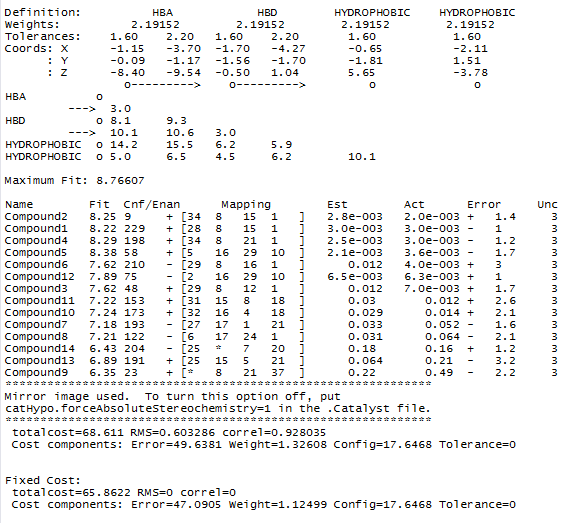


Null cost = 64.7

Tip-based liquid handling with serial dilution


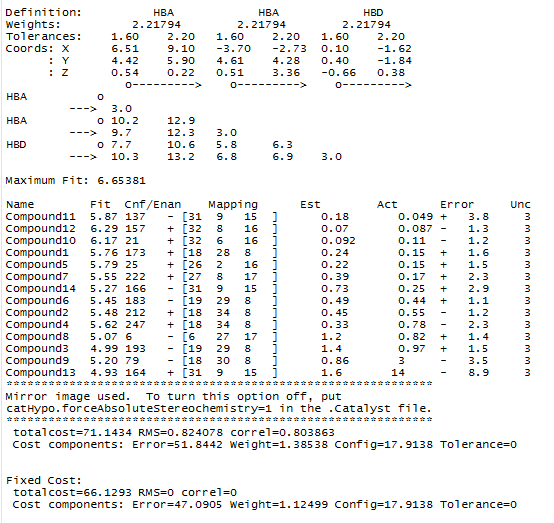


Null cost = 59.54
