# Supplementary material for: Dispensing Processes Impact Apparent Biological Activity as Determined by Computational and Statistical Analyses
Source: PLoS One. 2013 May 1;8(5):e62325. doi: 10.1371/journal.pone.0062325 (PMC3641061; doi:10.1371/journal.pone.0062325)
Supplement: Table S1 — Molecule structures, IC50 data and descriptors. (PDF) [file pone.0062325.s003.pdf]

#	Structure	MW	logP	H bond donors	H bond acceptors	SMILES	Molar refractivity (40-130)	Polar surface area <140 Å^2	LogD, pH 7	IC50 Echo = A	IC50 Tecan = B	log echo	log tecan	B/A	Charge at pH 7	pI
10	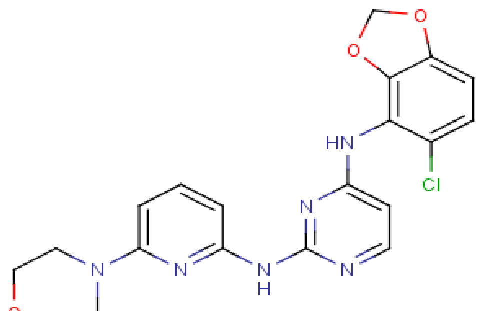	426.86	4.29	2	9	ClC1=CC=C2OCOC2=C1NC1=CC=NC(NC2=NC(=CC=C2)N2CCOCC2)=N1	112.5	93.66	4.23	0.064	0.817	-1.19382	-0.08778	12.76563	0.12	8.23
11	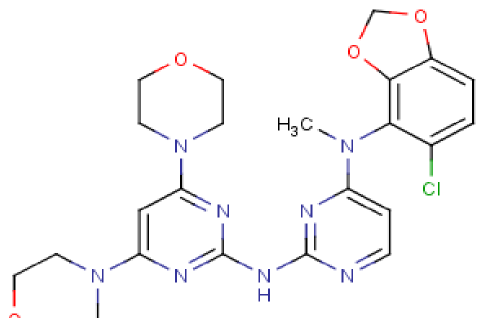	526.975	4.38	1	12	CN(C1=CC=NC(NC2=NC(=CC(=N2)N2CCOCC2)N2CCOCC2)=N1)C1=C2OCOC2=CC=C1Cl	139.57	110.23	4.01	0.486	3.03	-0.31336	0.481443	6.234568	0.36	7.5
4	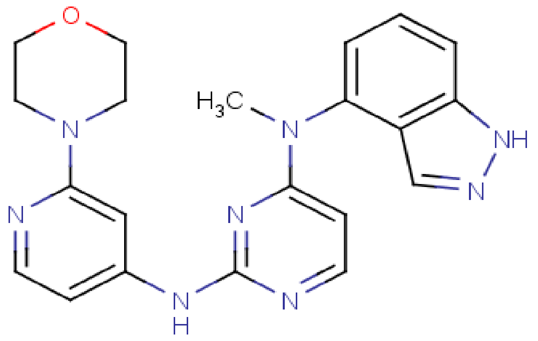	402.45	3.02	2	9	CN(C1=CC=NC(NC2=CC=NC(=C2)N2CCOCC2)=N1)C1=CC=CC2=C1C=NN2	116.53	95.09	1.73	0.003	0.146	-2.52288	-0.83565	48.66667	0.98	10.69
5	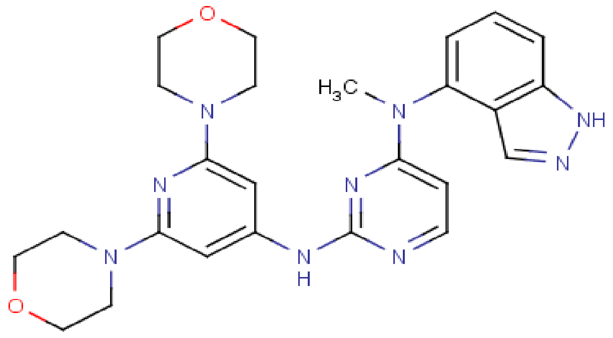	487.56	3.5	2	11	CN(C1=CC=NC(NC2=CC(=NC(=C2)N2CCOCC2)N2CCOCC2)=N1)C1=CC=CC2=C1C=NN2	140.34	107.56	1.6	0.002	0.553	-2.69897	-0.25727	276.5	1	11.19
6	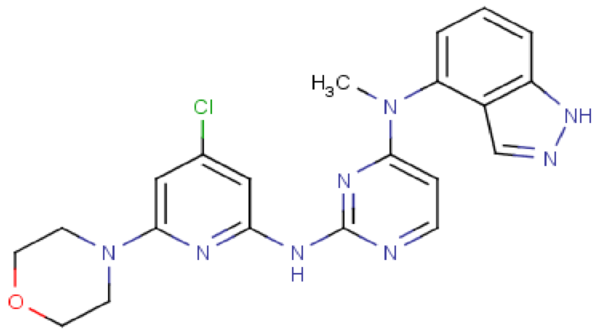	436.9	4.21	2	9	CN(C1=CC=NC(NC2=NC(=CC(Cl)=C2)N2CCOCC2)=N1)C1=CC=CC2=C1C=NN2	121.64	95.09	4.21	0.007	0.973	-2.1549	-0.01189	139	1.01	9.86
7	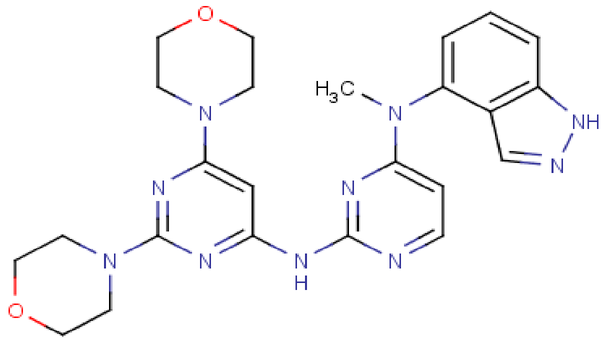	488.54	3.47	2	12	CN(C1=CC=NC(NC2=NC(=NC(=C2)N2CCOCC2)N2CCOCC2)=N1)C1=CC=CC2=C1C=NN2	139.11	120.45	3.47	0.003	0.778	-2.52288	-0.10902	259.3333	0.01	7.42

8	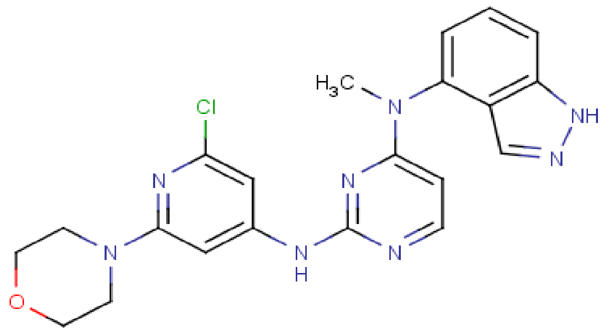	436.9	3.84	2	9	CN(C1=CC=NC(NC2=CC(=NC(Cl)=C2)N2CCOCC2)=N1)C1=CC=CC2=C1C=NN2	122.39	95.09	3.83	0.004	0.445	-2.39794	-0.35164	111.25	0.01	8.75
9	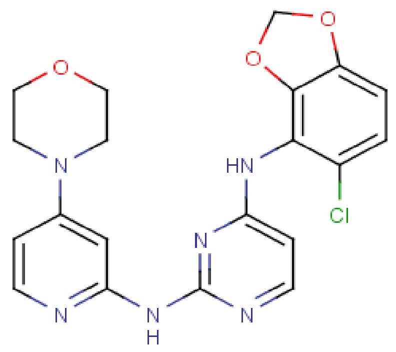	426.86	3.59	2	9	ClC1=CC=C2OCOC2=C1NC1=CC=NC(NC2=NC=CC(=C2)N2CCOCC2)=N1	112.19	93.66	2.57	0.052	0.17	-1.284	-0.76955	3.269231	0.96	9.27
W1	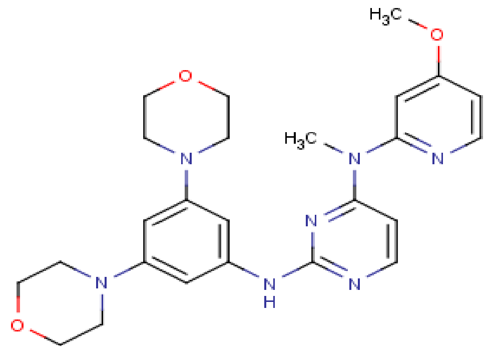	477.56	3.42	1	10	COC1=CC(=NC=C1)N(C)C1=NC(NC2=CC(=CC(=C2)N2CCOCC2)N2CCOCC2)=NC=C1	136.48	88.11	3.34	0.01362	0.112	-1.86582	-0.95078	8.223201	0.36	9.99
W11	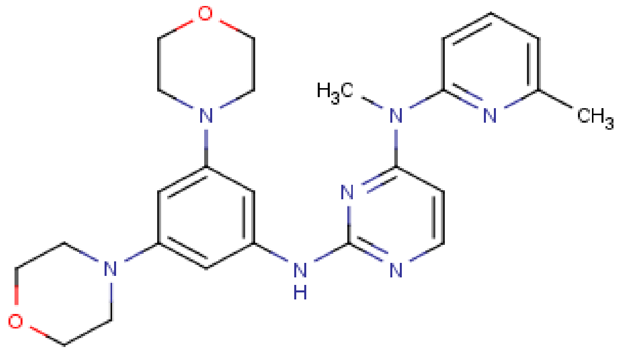	461.56	3.71	1	9	CN(C1=NC(C)=CC=C1)C1=NC(NC2=CC(=CC(=C2)N2CCOCC2)N2CCOCC2)=NC=C1	134.61	78.88	3.69	0.207	>14.4	-0.68403	#VALUE!	>69.565	0.05	9.67
W12	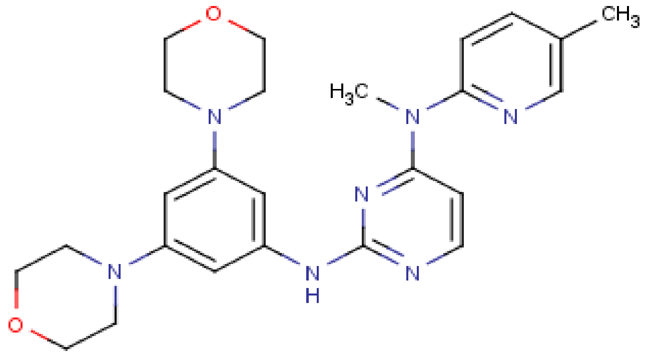	461.56	4.1	1	9	CN(C1=NC=C(C)C=C1)C1=NC(NC2=CC(=CC(=C2)N2CCOCC2)N2CCOCC2)=NC=C1	135.05	78.88	4.08	0.158	0.25	-0.80134	-0.60206	1.582278	0.02	9.52
W3	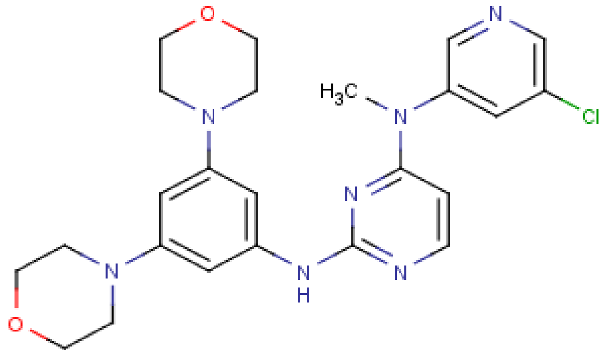	481.98	3.59	1	9	CN(C1=CC(Cl)=CN=C1)C1=NC(NC2=CC(=CC(=C2)N2CCOCC2)N2CCOCC2)=NC=C1	134.5	78.88	3.59	0.01164	0.049	-1.93405	-1.3098	4.209622	0.01	9.36

W5	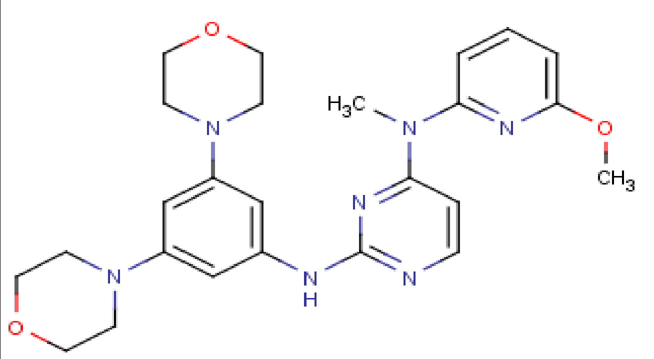	477.56	4.02	1	10	COC1=CC=CC(=N1)N(C)C1=NC(NC2=CC(=CC(=C2)N2CCOCC2)N2CCOCC2)=NC=C1	136.79	88.11	4.02	0.00633	0.087	-2.1986	-1.06048	13.74408	0.01	9.21
W7b	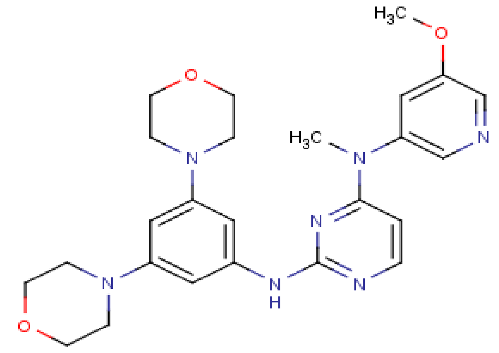	477.56	2.83	1	10	COC1=CC(=CN=C1)N(C)C1=NC(NC2=CC(=CC(=C2)N2CCOCC2)N2CCOCC2)=NC=C1	136.16	88.11	2.82	0.00358	0.152	-2.44612	-0.81816	42.4581	0.01	9.42
W081	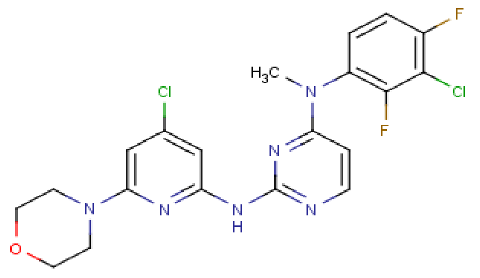	467.299	5.78	1	7		116.87	66.41	5.77		38.3		1.583199		1.01	9.85
W082.1	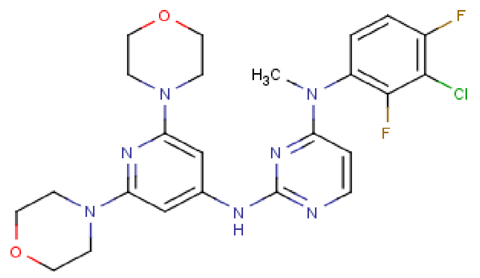	517.959	5.07	1	9		135.56	78.88	3.17		1.12		0.049218		1.01	11.22
W082.2	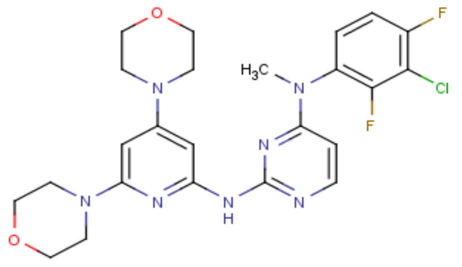	517.959	5.07	1	9		135.56	78.88	3.26		0.808		-0.09259		1	9.8
WO82.3	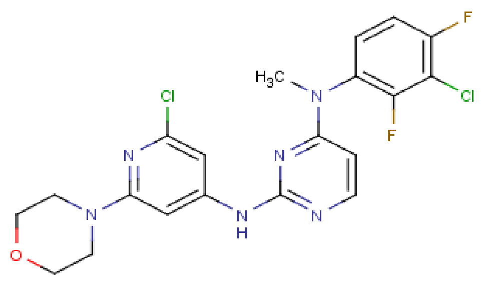	467.299	5.41	1	7		117.61	66.41	5.4		1.78		0.25042		0.02	8.83

WO82.4	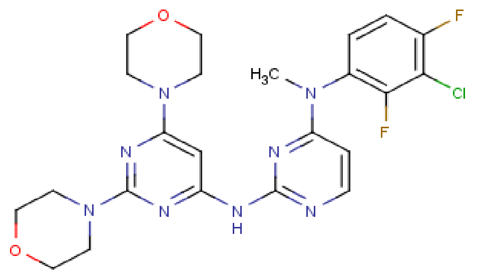	518.947	5.04	1	10		134.33	91.77	5.03		6.27		0.797268		0.01	7.4
WO83	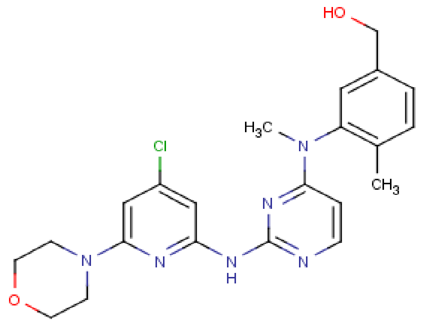	440.926	4.64	2	8		123.49	86.64	4.63		0.198		-0.70333		1.01	9.86
WO84.1	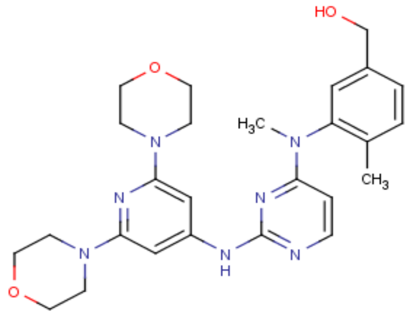	491.585	3.92	2	10		142.18	99.11	2.03		0.297		-0.52724		1	11.22
WO84.2	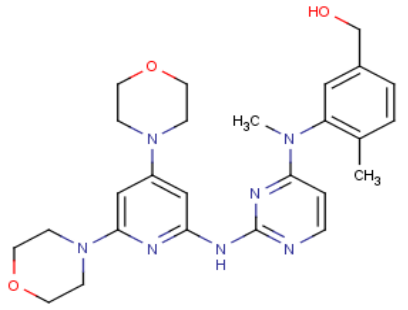	491.585	3.92	2	10		142.18	99.11	2.11		0.456		-0.34104		1	9.8
WO84.3	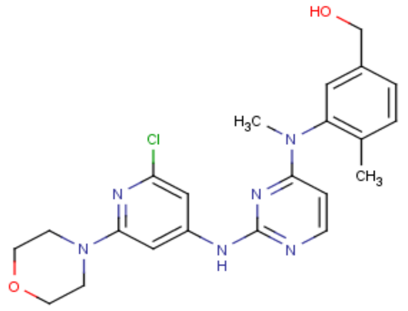	440.296	4.26	2	8		124.23	86.64	4.26		0.473		-0.32514		0.02	8.77
WO84.4	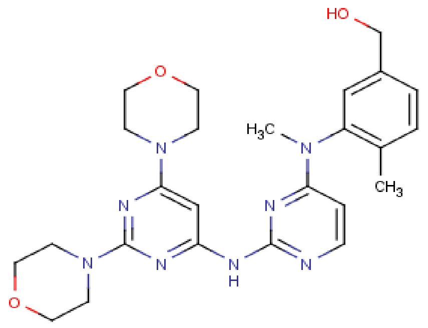	492.573	3.9	2	11		140.95	112	3.89		0.374		-0.42713		0.01	7.42

WO85.1	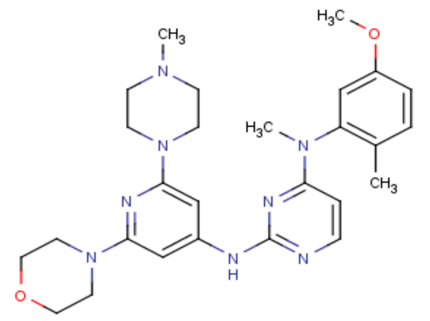	504.627	4.6	1	10		148.81	82.12	2.37	0.00231		-2.63639			1.74	10.88
WO85.2	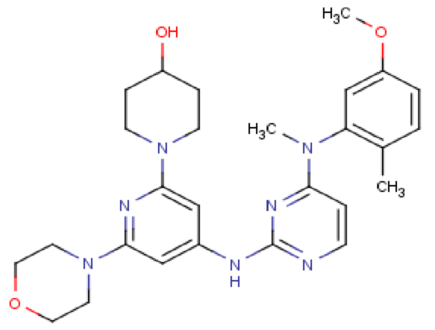	505.612	4.07	2	10		146.48	99.11	2.17	0.00325		-2.48812			1	11.24

Legend:
Simple numbers (e.g., 10) refer to compounds in US Patent 7,718,653, “Pyrimidine derivatives for inhibiting Eph receptors” (Barlaam, Ducray, & Kettle – AstraZeneca, Sodertalje, Sweden)

Numbers beginning with W (e.g., W7b) refer to compounds in WO 2009/010794 Al, "2,4-DIAMINO-PYRIMIDINE DERIVATIVES" (Barlaam & Ducray – AstraZeneca, Sodertalje, Sweden)

Numbers beginning with WO8 (e.g., WO82.1) refer to compounds in WO 2008/132505 Al "N' - (PHENYL) -N-(MORPHOLIN-4-YL-PYRIDIN-2-YL) -PYRIMIDINE-2, 4-DIAMINE DERNATIVES AS
EPHB4 KINASE INHIBITORS FOR THE TREATMENT OF PROLIFERATNE CONDITIONS" (Barlaam & Ducray – AstraZeneca, Sodertalje, Sweden)
