# Supplementary material for: Dispensing Processes Impact Apparent Biological Activity as Determined by Computational and Statistical Analyses
Source: PLoS One. 2013 May 1;8(5):e62325. doi: 10.1371/journal.pone.0062325 (PMC3641061; doi:10.1371/journal.pone.0062325)
Supplement: Table S2 — Test set data for searching with ‘acoustic dispensing’ pharmacophore. (DOCX) [file pone.0062325.s004.docx]

**Supplemental Data**

**Dispensing Processes Impact Apparent Biological Activity as Determined by Computational and Statistical Analyses**

*Sean Ekins^*1^, Joe Olechno^2^ and Antony J. Williams^3^*

^1^ Collaborations in Chemistry, 5616 Hilltop Needmore Road, Fuquay-Varina, NC 27526, U.S.A.

^2^ Labcyte Inc., 1190 Borregas Avenue, Sunnyvale, CA 94089, U.S.A.

^3^ Royal Society of Chemistry, 904 Tamaras Circle, Wake Forest, NC 27587, U.S.A.

**Table S2.** Test set data for searching with ‘acoustic dispensing’ pharmacophore – data ranked by predicted value. ND = no experimental data.

| \| **Name** \| **Acoustic IC_50_ Prediction (µM)** \| \| **Tip-based IC_50_ Actual (µM)** \| **Acoustic IC_50_ Actual (µM)** \| \| \| --- \| --- \| --- \| --- \| --- \| --- \| \| W085.1 \| 0.0196 \| ND \| \| 0.00231 \| \| W084.1 \| 0.025542 \| 0.297 \| \| ND \| \| W085.2 \| 0.026467 \| ND \| \| 0.00325 \| \| W082.2 \| 0.033615 \| 0.808 \| \| ND \| \| W084.4 \| 0.060011 \| 0.374 \| \| ND \| \| W084.2 \| 0.0761 \| 0.456 \| \| ND \| \| W084.3 \| 3.38573 \| 0.473 \| \| ND \| \| W083 \| 3.84933 \| 0.198 \| \| ND \| \| W081 \| 5.47847 \| 38.3 \| \| ND \| \| W082.3 \| 7.49263 \| 1.78 \| \| ND \| \| W082.1 \| 7.82218 \| 1.12 \| \| ND \| \| W082.4 \| 12,622.20 \| 6.27 \| \| ND \| | | | |  |  |  |
| --- | --- | --- | --- | --- | --- | --- | --- | --- | --- | --- | --- | --- | --- | --- | --- | --- | --- | --- | --- | --- | --- | --- | --- | --- | --- | --- | --- | --- | --- | --- | --- | --- | --- | --- | --- | --- | --- | --- | --- | --- | --- | --- | --- | --- | --- | --- | --- | --- | --- | --- | --- | --- | --- | --- | --- | --- | --- | --- | --- | --- | --- | --- | --- | --- | --- | --- | --- | --- | --- | --- | --- | --- |
|  | | | |  |  |  |
|  |  |  |  |  |  |  |
|  |  |  |  |  |  |  |
