# Supplementary material for: Dispensing Processes Impact Apparent Biological Activity as Determined by Computational and Statistical Analyses
Source: PLoS One. 2013 May 1;8(5):e62325. doi: 10.1371/journal.pone.0062325 (PMC3641061; doi:10.1371/journal.pone.0062325)
Supplement: Table S3 — Test set data for searching with ‘tip-based dispensing’ pharmacophore. (DOCX) [file pone.0062325.s005.docx]

**Supplemental Data**

**Dispensing Processes Impact Apparent Biological Activity as Determined by Computational and Statistical Analyses**

*Sean Ekins^*1^, Joe Olechno^2^ and Antony J. Williams^3^*

^1^ Collaborations in Chemistry, 5616 Hilltop Needmore Road, Fuquay-Varina, NC 27526, U.S.A.

^2^ Labcyte Inc., 1190 Borregas Avenue, Sunnyvale, CA 94089, U.S.A.

^3^ Royal Society of Chemistry, 904 Tamaras Circle, Wake Forest, NC 27587, U.S.A.

**Table S3**. Test set data for searching with ‘tip-based dispensing’ pharmacophore– data ranked by predicted value. Note W081 and W082.3 were not retrieved by the pharmacophore indicating they do not fit to the Tip-based pharmacophore. These correspond to IC_50_ values of 38.3 μM and 1.78 μM, respectively. ND = no experimental data.

| **Name** | **Tip-based IC_50_ Prediction (µM)** | **Tip-based IC_50_ Actual (µM)** | **Acoustic IC_50_ Actual (µM)** |
| --- | --- | --- | --- |
| W084.1 | 0.348797 | 0.297 | ND |
| W084.2 | 0.380588 | 0.456 | ND |
| W084.4 | 0.699411 | 0.374 | ND |
| W082.2 | 0.839169 | 0.808 | ND |
| W085.2 | 1.32573 | ND | 0.00325 |
| W082.4 | 1.49893 | 6.27 | ND |
| W083 | 2.82285 | 0.198 | ND |
| W084.3 | 2.91191 | 0.473 | ND |
| W082.1 | 3.38289 | 1.12 | ND |
| W085.1 | 3.54739 | ND | 0.00231 |
